# Roles of Fatty Acids in Microglial Polarization: Evidence from In Vitro and In Vivo Studies on Neurodegenerative Diseases

**DOI:** 10.3390/ijms23137300

**Published:** 2022-06-30

**Authors:** Sanjay, Miey Park, Hae-Jeung Lee

**Affiliations:** 1 Department of Food Science and Biotechnology, College of BioNano Technology, Gachon University, Seongnam-si 13120, Korea; sanjay.monga4@gmail.com; 2 Department of Food and Nutrition, College of BioNano Technology, Gachon University, Seongnam-si 13120, Korea; 3 Institute for Aging and Clinical Nutrition Research, Gachon University, Seongnam-si 13120, Korea

**Keywords:** neuroinflammation, neurodegeneration, fatty acids, microglial polarization, M1/M2 phenotype, microglial modulation, brain macrophages, polyunsaturated fatty acids, apoptosis, antioxidation

## Abstract

Microglial polarization to the M1 phenotype (classically activated) or the M2 phenotype (alternatively activated) is critical in determining the fate of immune responses in neurodegenerative diseases (NDs). M1 macrophages contribute to neurotoxicity, neuronal and synaptic damage, and oxidative stress and are the first line of defense, and M2 macrophages elicit an anti-inflammatory response to regulate neuroinflammation, clear cell debris, and promote neuroregeneration. Various studies have focused on the ability of natural compounds to promote microglial polarization from the M1 phenotype to the M2 phenotype in several diseases, including NDs. However, studies on the roles of fatty acids in microglial polarization and their implications in NDs are a rare find. Most of the studies support the role of polyunsaturated fatty acids (PUFAs) in microglial polarization using cell and animal models. Thus, we aimed to collect data and provide a narrative account of microglial types, markers, and studies pertaining to fatty acids, particularly PUFAs, on microglial polarization and their neuroprotective effects. The involvement of only PUFAs in the chosen topic necessitates more in-depth research into the role of unexplored fatty acids in microglial polarization and their mechanistic implications. The review also highlights limitations and future challenges.

## 1. Introduction

Neurodegenerative diseases (NDs) are characterized by the progressive loss of the structure or function of neurons in the central nervous system (CNS). Alzheimer’s disease (AD), Parkinson’s disease (PD), Huntington’s disease (HD), multiple sclerosis (MS), cerebral ischemia, and amyotrophic lateral sclerosis (ALS) are typical NDs. Although various NDs exhibit differential pathology, neuroinflammation is one of the pathological factors that contribute to the progression of all NDs [1].

### 1.1. Neuroinflammation and Neurodegeneration

Neuroinflammation is a complex immune defense system used by the human body to respond to external stimuli in the CNS. All CNS cells, mainly microglia and astrocytes, are activated in response to inflammatory stimuli and subsequently release several pro-inflammatory cytokines. These cells return to their physiological state after the alleviation of the infection owing to the activity of anti-inflammatory cytokines [2,3]. Under physiological conditions, astrocytes and neurons regulate the activation of microglial cells. However, pathological conditions, such as cell death or neuronal damage, impair the ability of astrocytes and neurons to regulate microglial cell activation. Microglial cell-mediated neuroinflammation is an important pathological feature of NDs [4,5]. Several studies have suggested that inflammatory pathways play an essential role in the pathogenesis of NDs [6].

### 1.2. Microglia: A Double-Edged Sword

**Figure 1:**
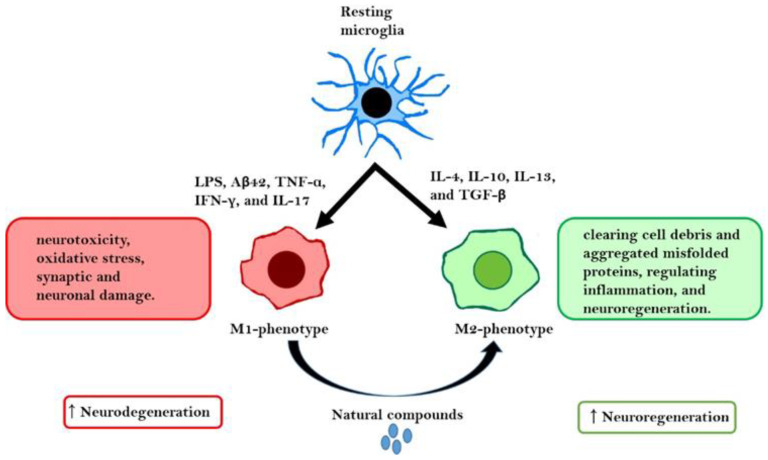
Microglial cells as a double-edged sword. Resting microglial cells can polarize into the M1 or M2 phenotype depending on the external stimulus. Lipopolysaccharide (LPS), amyloid-beta (Aβ), tumor necrosis factor-α (TNF-α), interferon-gamma (IFN-γ), and interleukin (IL)-17 are reported to promote microglial polarization toward the M1 phenotype, which promotes neurodegeneration. In contrast, IL-4, IL-10, IL-13, and transforming growth factor-β (TGF-β) promote microglial polarization toward the M2 phenotype, which exerts neuroprotective effects and promotes neuroregeneration. Natural compounds can promote the polarization of the M1 phenotype to the M2 phenotype.

Microglial cells, which are the resident brain macrophages, account for approximately 5–12% of all CNS-specific cells. In other anatomic regions, microglial cells exhibit morphological heterogeneity. Microglial cells originate from myeloid precursor cells in the embryonic yolk sac and migrate to the brain parenchyma through blood circulation during CNS development [7,8]. The characteristics of microglial cells, which are involved in neuroprotection and surveillance, include the ability to migrate, sense the extracellular environment, phagocytose foreign material and cellular debris, and clear pathogens [9]. The resting state or non-activated microglial cells (M0-type, which have not encountered any foreign material) exhibit diverse morphologies and long extensions that surround the neurons and other cells. Additionally, M0 microglial cells survey the local environment and are activated or tend to retract and exhibit amoeboid morphology upon encountering foreign materials, such as lipopolysaccharide (LPS), cell debris, blood–brain barrier (BBB) leakage, or damaged cells. Morphological changes play a vital role in microglial function, as they contribute to the migration of microglial cells toward lesion sites [10,11,12]. M1 microglial cells, also known as classically activated microglia, are activated upon encountering pathogens or extracellular stress. The M1 phenotype is associated with an inflammatory response that contributes to neurotoxicity, oxidative stress, and neuronal and synaptic damage. M1 microglial cells serve as the first line of defense against invading pathogens. This is followed by the activation of alternatively activated microglial cells, which exert an anti-inflammatory effect, regulate neuroinflammation by clearing cell debris and misfolded proteins, and promote neuroregeneration [13,14]. A pictorial representation of microglial polarization is shown in Figure 1.

### 1.3. Microglial Polarization as an Inflammation/Anti-Inflammation Switch

The M1 phenotype is primarily activated by interferon-gamma (IFN-γ), while the M2 phenotype is activated by interleukin (IL)-4-dependent immune responses [15]. The M1/M2 nomenclature was first used for purified stimuli under in vitro conditions. The suggested nomenclature for M1/M2 was “M1-like” and “M2-like phenotype” based on the gene expression profile [16,17]. The phenotypes of microglial cells, which exhibit plasticity, can be switched depending on the cytokine changes in the environment [18]. The M2 phenotype of microglia is further classified into four different subtypes (M2a, M2b, M2c, and newly identified M2d) based on their specific functions. For example, regeneration and repair are mediated by the M2a subtype. The M2b subtype exists in a transition state and is involved in the immune response, whereas the M2c subtype is involved in the release of anti-inflammatory cytokines and exerts neuroprotective effects [19,20]. M2d macrophages, also known as tumor-associated macrophages, are involved in tumor angiogenesis [21]. The phenotypic differences between M1 and M2 microglial cells are determined by the differential expression of distinct cell-surface receptors and the production of mediators with specific functions. Although classically activated microglial cells play a crucial role in preventing the accumulation of aberrant proteins or cell debris by releasing inflammatory mediators, aberrant activation of these microglial cells may promote neuroinflammatory reactions that affect different regions of the CNS. Excessive activation of M1 microglial cells and the dysfunction of M2 microglial cells markedly promote the development of NDs [22].

The phenotypes, specific cytokines, chemokines, markers, and functions of microglia are listed in Table 1.

In addition, using bulk and single-cell RNA sequencing, transcriptomic analysis revealed different transcription states of microglia in different regions of the CNS [28,29,30,31,32,33]. Microglia show different characteristic genes during developmental processes, disease, or aging, such as disease-associated microglia (DAM), plaque-associated microglia (PAM) [34,35,36], injury-responsive microglia (IRM) [37], microglial neuroregeneration (MGnD) phenotype [38], activated response microglia (ARM) [39], white matter-associated microglia (WAM) [40], proliferative-region-associated microglia (PAM) [41], axon tract-associated microglia (ATM) [37], interferon response microglia (IRM) [39], and lipid-droplet-accumulating microglia (LDAM) [42]. It was found that microglia downregulate homeostatic genes and upregulate phagocytosis, lysosomal development, and lipid metabolism-related genes in pathological conditions. Although researchers are unable to explore the differences between these different states, a set of genes that are primarily expressed in these states can display some similarities.

DAM1 and DAM2 are two microglial states reported as DAM sub-populations by Karen-Shaul et al. Both of their genes are induced in a TREM2-dependent or -independent manner [43]. Moreover, the highly expressed genes in DAM2, namely, apolipoprotein E (Apoe), lysozyme 2 (Lyz2), cathepsin B (Ctsb), C-type lectin domain family 7 member A (Clec7a), secreted phosphoprotein 1 (Spp1), galectin-3 (Lgals3), integrin subunit alpha X (Itgax), lipoprotein lipase (Lpl), cystatin F (Cst7), glycoprotein Nmb (Gpnmb), ferritin heavy chain 1 (Fth1), insulin-like growth factor 1 (Igf1), and cathepsin D (Ctsd), are upregulated in MGnD, plaque-associated microglia, and ARM, thereby suggesting similar types of microglia [38,39,43].

The white matter of an aged mouse brain was found to possess WAM, which express genes related to DAM2. However, the major difference between the two is the upregulation of myeloid cell surface antigen CD33, SLIT-ROBO Rho GTPase-activating protein 2 (Srgap2), and ATP binding cassette subfamily A member 1 (Abca1), which are upregulated in WAM but not in DAM2. In contrast, TYRO protein tyrosine kinase-binding protein (Tyrobp) and macrophage migration inhibitory factor (Mif) are upregulated in DAM2 but not in WAM [40]. Furthermore, the DAM/WAM signature is expressed in ATM and proliferative-region-associated microglia and transiently detected in the white matter of a developing mouse brain [41,44,45,46].

IRM (IR 2.2) express interferon responses such as IFN-induced protein with tetratricopeptide repeats 1 (Ifit1) and C-X-C motif chemokine ligand 10 (Cxcl10), whereas IR2.3 express cytokines such as chemokine (C-C motif) ligand 3 (Ccl3) and interleukin 1 beta (Il1b), thus suggesting the presence of microglia specialized to express IFN response genes or inflammatory cytokines, in addition to DAM-related states [37].

Two microglial states were found to be present in an amyloid precursor protein (APP) knock-in mouse model of Alzheimer’s disease (AD): ARM, which is similar to DAM and IFN response microglia expressing Ifit2, Ifit3, IFN regulatory factor 7 (Irf7), IFN-induced transmembrane protein 3 (Ifitm3), and 2′-5′ oligoadenylate synthetase-like 2 (Oasl2) [39].

In the neuroinflammatory state, DAM induced in EAE can be seen in three different subsets using Cxcl10, cluster of differentiation 74 (Cd74), and Ccl5 [28]. Cd74-low DAM are highly proliferative and less likely to interact with encephalitogenic T cells than Cd75-high DAM, which suggests the role of specific clusters of DAM in providing functional differences. However, the molecular mechanism behind different microglial states and functional differences in aging, development, and disease need further in-depth research.

Therefore, the modulation of microglial polarization is a potential therapeutic strategy for NDs. Recent studies have analyzed specific natural or synthetic compounds that can modulate M1/M2 microglial polarization and suggested that they can serve as potential therapeutic agents for NDs. Furthermore, studies documenting the impact of the microbiome on microglial polarization have also been reported [47,48,49].

### 1.4. Fatty Acids and Their Bioactive Potential

**Figure 2:**
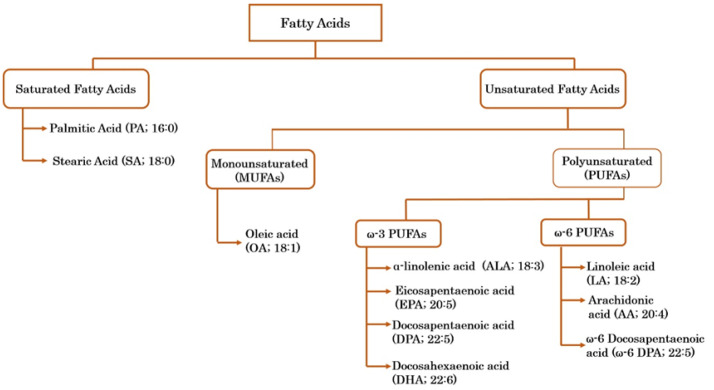
Fatty acids are classified into the following two main categories: saturated and unsaturated fatty acids. Saturated fatty acids are palmitic acid and stearic acid. Unsaturated fatty acids are further classified into monounsaturated and polyunsaturated fatty acids. Oleic acid with one double bond is a monounsaturated fatty acid. Polyunsaturated fatty acids are sub-divided into the following groups: ω-3 fatty acids (α-linolenic acid, eicosapentaenoic acid, docosapentaenoic acid, and docosahexaenoic acid) and ω-6 fatty acids (linoleic acid, arachidonic acid, and docosapentaenoic acid).

Fatty acids, which are dietary components with essential structural, metabolic, and physiological functions in the human body, are a primary energy source and serve as precursors for signaling molecule synthesis [50]. Additionally, fatty acids supply essential components of the cell membrane. Fatty acids can be classified into different categories depending on the number of carbon atoms and double bonds. Briefly, fatty acids are classified as saturated fatty acids (SFAs), monounsaturated fatty acids (MUFAs), and polyunsaturated fatty acids (PUFAs). PUFAs are further sub-classified into omega (ω)-3 and ω-6 PUFAs depending on the location of the first double bond. The first double bond in ω-3 PUFAs is at the third carbon atom, whereas that in ω-6 PUFAs is at the sixth carbon atom from the methyl end [51]. Fatty acids and their classifications are shown in Figure 2.

Previous studies have reported that fatty acids are potent therapeutic agents for cardiovascular diseases, diabetes, depression, NDs, pediatric disorders, and cancer [52,53,54]. Additionally, fatty acids exhibit antioxidative and anti-inflammatory properties [55,56,57]. Nadjar et al. [58] reviewed fatty acid-mediated microglial modulation. However, to the best of our knowledge, the role of fatty acids in microglial polarization in NDs has not been previously reviewed.

## 2. Literature Search Strategy

A literature search was performed using Google Scholar, PubMed, and Science Direct repositories to retrieve published studies; the following keywords were used to find the relevant information on the role of fatty acids in the modulation of microglial polarization in NDs: “fatty acids in microglial polarization” or “fatty acids and M1/M2 shift” or “role of fatty acids in neurodegenerative diseases” or “natural compounds and microglial polarization” or “effects of fatty acids in neurodegenerative diseases” or “Anti-inflammatory effects of fatty acids in neurodegeneration” or “Neuroinflammation and Microglial polarization”. We identified 28 studies showing the potential of fatty acids in modulating microglial polarization. Exclusion/inclusion criteria were applied after finding out whether the studies showed the potential effects of fatty acids on the expression of M1/M2 specific cytokines and markers in neurodegenerative disease models.

## 3. Role of Fatty Acids in Microglial Polarization in NDs

Lipids are considered vital biochemical factors of the brain and are known to perform many different functions. The composition of lipids varies widely, and their composition, metabolism, and signaling play essential roles in neuropsychiatric, neurodevelopmental, and neurodegenerative disorders [59]. PUFAs from the n-6 and n-3 families are lipids that rely on dietary supply and are considered crucial for brain development [59,60,61]. In addition to providing the structure and function of cell membranes, n-3 and n-6 PUFAs are substrates for the production of several signaling molecules involved in the physiological function of cells, especially in the brain [62]. In recent years, PUFAs have gained scientists’ attention, as they were found to modulate microglial activity and neuroinflammation. Bioactive fatty acids derived from nutrition, such as SFAs and PUFAs, can cross the blood–brain barrier and directly influence microglia, as recently reviewed [62]. n-6 and n-3 PUFAs regulate neuroinflammation either directly or through their metabolites, such as oxylipins or endocannabinoids [63]. The role of dietary PUFAs in microglial development has been poorly researched, but there are findings available that define the influence of dietary PUFAs on microglial lipid composition [63,64]. Moreover, microglia were found to display a unique fatty acid profile with EPA enrichment, pointing at cells as a source of EPA-derived oxylipins with anti-inflammatory activities [64]. Indeed, resolvin D1 (RvD1) and resolvin E1 (RvE1), which are pro-resolutive oxylipins derived from DHA and EPA, respectively, reduced pro-inflammatory cytokine expression triggered in microglia by LPS in vitro [65].

Some of the possible mechanisms through which PUFAs show anti-inflammatory activities or modulate microglial phenotypes are the activation and overexpression of the protein deacetylase sirtuin1 (SIRT1), with subsequent suppression of NF-κB via subunit p65 deacetylation [66]. Inhibition of the p38MAPK inflammatory pathway and PPAR-γ activation are also in part responsible for the protective effects of PUFAs and their products [67,68], given the connection of NF-κB and PPAR-γ to the bioenergetic state of microglia [69]. In addition, dietary lipids may likely shape the microglia phenotype by acting on cell metabolism. In support of this hypothesis, fasting and the ketogenic diet, which lead to a sustained reduction in blood glucose levels and to an increase in circulating ketones, have been reported to have anti-inflammatory actions and suppress the activation of microglia by regulating their metabolic features [70]. These effects have been shown to rely on the activation of the metabolite receptor GPR109A by B-hydroxybutyrate, which attenuates NF-κB signaling and pro-inflammatory cytokine production [71].

NDs, such as AD, PD, ALS, MS, and HD, are characterized by neuronal degeneration in specific regions of the CNS and share common pathophysiological mechanisms, including inflammation and aberrant protein deposition [23,72]. The current review summarizes some of the reported mechanisms through which PUFAs are able to modulate different states of microglia in NDs.

### 3.1. Role of Fatty Acids in Microglial Polarization in Neuroinflammation

The term neuroinflammation refers to the complex immune mechanism underlying the CNS response to pathological stimuli through the activation of microglia astrocytes and local immune cell invasion by producing pro-inflammatory mediators, which reverts to normal with the help of endogenous anti-inflammatory mediators [2,3]. Microglial cells are the major cellular components of the CNS and mediate neuroinflammation in NDs [4,5]. Under physiological conditions, astrocytes and neurons regulate microglial activation [6]. However, astrocytes and neurons cannot optimally regulate microglial activation under pathological conditions, including cell death or neuronal degeneration. Neuroinflammation also impairs the ability of the brain to generate new neurons [73]. Thus, neuroinflammation can be a potential therapeutic target for NDs. Several therapeutic approaches can be employed to mitigate the deleterious effects of neuroinflammation.

The mitogen-activated protein kinase (MAPK) pathway comprises a highly conserved family of serine/threonine kinases, including extracellular signal-regulated kinase 1/2 (ERK1/2), p38, and c-Jun N-terminal kinase (JNK) isoforms. MAPK signaling regulates inflammatory responses and promotes macrophage/microglial polarization toward the M2 phenotype [74]. Docosahexaenoic acid (DHA) is reported to regulate MAPK signaling and exhibit anti-inflammatory and anti-neurogenic activities in LPS-induced primary microglial cells [67]. Additionally, DHA markedly inhibited the expression levels of nitric oxide (NO) and inducible nitric oxide synthase (iNOS) in primary glial cultures (cerebral cortex of rats). Furthermore, DHA downregulated the expression levels of M1-specific cytokines (Table 2) and mitigated the LPS-induced downregulation of insulin-like growth factor (IGF)-1 expression, induced peroxisome proliferator-activated receptor-γ (PPAR-γ) nuclear translocation, enhanced neural cell progenitor cell (NPC) survival, and promoted NPC differentiation.

In 2014, Madore et al. reported the importance of ω-3 PUFAs in neurodevelopment [75]. C57BL6/J and CX3CR1-eGFP mice were fed a ω-3 PUFA diet or a ω-3 PUFA-deficient diet after mating (Table 2). ω-3 PUFA deficiency decreased the brain fatty acid levels, especially ω-3 PUFAs, in the developing brain and increased the levels of pro-inflammatory cytokines, such as IL-1β and IL-6. Additionally, ω-3 PUFA deficiency upregulated the expression levels of the M1 marker cyclooxygenase-2 (COX-2) but downregulated those of M2-specific markers, such as cluster of differentiation 36 (CD36) and CD206, during birth. In contrast, the expression levels of M1 and M2 markers were downregulated at weaning. Additionally, ω-3 PUFA deficiency decreased microglial motility and neuronal plasticity, as evidenced by the upregulation of Erg1 and the downregulation of c-Jun and brain-derived neurotrophic factor (BDNF). Ruiz-Roso et al. provided in vitro evidence to support the role of ω-3 fatty acids in modulating the microglial phenotype [76]. The authors pretreated BV-2 microglial cells with DHA with 50 and 500 ppm of phytanic acid (PhA). Pretreatment with DHA (with 50 ppm of PhA) attenuated LPS-induced or hydrogen peroxide (H2O2)-induced neuroinflammation (Table 2). Compared with those in the LPS-treated or H2O2-treated group, the levels of BV-2 cell death, lactate dehydrogenase (LDH) secretion, cleaved caspase-3 (a marker of cell death) expression, and oxidative O2− ion concentration were lower. Additionally, DHA promoted microglial polarization from the M1 phenotype to the M2 phenotype by downregulating COX-2, IL-6, iNOS, and CD11b and upregulating arginase-1 (Arg1) and the levels of the neurotrophic factor BDNF. Furthermore, the antioxidant activity of DHA with 50 ppm of PhA was lower than that of DHA with 500 ppm of PhA, which can be attributed to the antioxidative effects of PhA, as defined earlier [77,78].

A recent study also reported the neuroprotective effects of DHA against Japanese encephalitis virus (JEV) infection-induced neuroinflammation [79]. JEV elicits an inflammatory response and induces neuronal loss upon invasion of the BBB and CNS [80,81]. JEV-infected primary neuron/glia, neuron, and microglial cell cultures were treated with DHA. DHA promoted microglial polarization from the M1 phenotype to the M2 phenotype and mitigated neuronal death and microglial morphology alterations. Additionally, DHA decreased the LDH efflux and inhibited the production of JEV particles, genomic RNA, and nonstructural protein 1 (NS1) (Table 2). Pro-inflammatory transcription factors, such as nuclear factor kappa-light-chain-enhancer of activated B cells (NF-κB), activator protein (AP)-1, and cAMP response element-binding protein (CREB), which are involved in pro-inflammatory cytokine expression and microglial polarization [82], were downregulated upon supplementation with DHA. Moreover, the Toll-like receptor (TLR)/IFN axis plays an essential role in the release of pro-inflammatory cytokines [83,84]. DHA exerted inhibitory effects on the expression of various molecules, such as TLR4, TLR7, myeloid differentiation primary response 88 (MyD88), IFN-regulatory factor 1 (IRF1), NLR family pyrin domain containing 3 (NLRP3), and apoptosis-associated speck-like protein containing a CARD (ASC), and the phosphorylation of transforming growth factor β-activated kinase-1 (TAK1), TANK-binding kinase (TBK1), IRF3, ERK, JNK, p38, serine/threonine-specific protein kinase (Akt), cytosolic phospholipase A2 (cPLA2), Janus kinase 1 (Jak1), Jak2, tyrosine kinase 2 (Tyk2), Src, signal transducers and activators of transcription 1 (Stat1), and Stat2. The effects of DHA, which were mediated through the G protein-coupled receptor (GPR120), in the pretreatment group were more favorable than those in the post-treatment group. One recent study [85] focused on the role of ω-3 PUFAs in microglial polarization in LPS-induced C56B1/6J mice (Table 2). DHA downregulated the expression of M1-specific markers, nerve growth factor (NGF), and LPS-induced TLR4, which is a common pattern recognition receptor that recognizes extracellular LPS [86].

The anti-inflammatory activities of N-docosahexaenoylethanolamine (synaptamide) and N-eicosapentaenoylethanolamine (EPEA), which are ethanolamide derivatives of DHA and eicosapentaenoic acid (EPA), respectively, were demonstrated in an in vivo inflammation model [87]. SIM-A9 microglial cells were pretreated with synaptamide and EPEA preparations and stimulated with LPS. Synaptamide and EPEA downregulated pro-inflammatory cytokines and upregulated anti-inflammatory cytokines. However, the results of experiments with LPS-treated C57BL/6 mice revealed that synaptamide (but not EPEA) downregulated IL-1β and tumor necrosis factor-α (TNF-α). EPEA and synaptamide upregulated the production of the anti-inflammatory cytokine IL-4. Meanwhile, only synaptamide upregulated the expression of IL-10. Synaptamide downregulated the expression of the M1-specific marker CD86 and major histocompatibility complex-II (MHC-II), whereas EPEA only suppressed the expression of MHC-II but not that of CD86. EPEA and synaptamide mitigated the LPS-induced downregulation of the M2-specific markers CD206 and Arg1. Additionally, synaptamide mitigated the LPS-induced upregulation of ionized calcium-binding adaptor molecule-1 (Iba1)-positive area within the hippocampus. EPEA and synaptamide downregulated the expression levels of glial fibrillary acidic protein (GFAP) and S100 calcium-binding protein (S100) β and increased long-term potentiation (LTP). Only synaptamide increased the BDNF levels (Table 2).

An in vitro study on BV-2 and SH-SY5Y cells by Liu et al. reported that ω-3 docosapentaenoic acid (DPA) exerted protective effects on LPS-induced neuroinflammation [88]. DPA attenuated LPS-induced cell death and downregulated the expression of M1-specific cytokines and markers (IL-1β, TNF-α, IL-1R, NO, Iba-1, and CD11b) and M2-specific cytokines and markers (IL-10, Arg1, and CD206) (Table 2).

**Table 2:** In vitro and in vivo studies on fatty acid-mediated microglial polarization in neuroinflammation.

Compound	Cell/Animal	Treatment	Findings	Ref
DHA	Primary microglial cultures	DHA (0.1, 1, 10, or 20 μM) + LPS (10 ng/mL) or IFN-γ (200 U/mL) for 24 h.	↓NO, ↓iNOS, ↓TNFα, ↓IL-6, ↓Arg1, ↓IL-10, ↓PGE2, ↓MAPK, ↑PPARγ nuclear translocation, and ↑NPC survival and differentiation.	[67]
ω-3 PUFAs	C57BL6/J and CX3CR1-eGFP	Sunflower oil (6% fat; rich in LA, ω-3 PUFA-deficient diet) or a mixture of different oils containing ALA (ω-3 PUFA diet).	↓ω-3 PUFAs, ↑IL-1β, ↑IL-6, ↑COX-2, ↓CD36 ↓CD206, ↓microglial motility, ↑Erg-1, ↓c-Jun, and ↓BDNF.	[75]
DHA +PhA	BV-2 cells	DHA containing 50 or 500 ppm of PhA for 1 h + LPS (0.1 µg/mL) or H_2_O_2_ (0.8 mM) for 24 h.	↑Cell viability, ↓LDH, ↓caspase-3, ↓O_2_^−^, ↓COX-2, ↓IL-6, ↓iNOS, ↓CD11b, ↑Arg1, ↓GtPx, ↓GtRd, ↓SOD-1, and ↑BDNF.	[76]
DHA	Primary neuron/glia, neuron, and microglial cultures (SD rats)	DHA (50 μM) for 3 h + JEV (MOI = 5) for 1 h + DHA (50 μM) for 3 h. JEV (MOI = 5) for 1 h + DHA (50 μM) for 48 h	↓Nitrite, ↓IL-1β, ↓TNFα, ↓PGE2, ↓ROS, ↓neuronal death, ↓phagocytic microglia, ↓LDH efflux, ↓JEV RNA, ↓NS1, ↓viral particle production, ↓CD68, ↓iNOS, ↓COX-2, ↓IRF5, ↓IRF8, ↓P2X4R, ↓P2X7R, ↓P2Y12R, ↑CD163, ↑CD206, ↑Arg1, ↑Nrf2, ↑HO-1, ↑miR-124, ↓NF-κB, ↓AP-1, ↓CREB, ↓TLR4, ↓TLR7, ↓MyD88, ↓IRF1, ↓NLRP3, ↓ASC, and ↓phosphorylation (TAK1, TBK1, IRF3, ERK, JNK, p38, Akt, cPLA2, Jak1, Jak2, Tyk2, Src, Stat1, and Stat2). Pretreatment > post-treatment.	[79]
DHA	C57Bl/6J mice	Fish hydrolysate (DHA; 143 µg) or DHA (10 mg) for 18 days + LPS (125 µg/kg bodyweight) for 2 h.	↓TLR4, ↓IL-1β, ↓TNFα, ↓IL-6, ↓CCL-2, ↓CD68, ↓CD11b, ↓CD86, ↓SOCS3, ↓NGF, and ↓COX-2.	[85]
Syn and EPEA	SIM-A9 cells, C57BL/6 mice	Syn or EPEA (10 µM) for 1 h + LPS (1 µg/mL) for 24 h. Syn or EPEA (100 mg/mL) + LPS (750 mg/kg bodyweight), injection/day for 7 days.	(Syn): ↓IL-1β, (Syn and EPEA): ↓TNFα, ↓IL-6, and ↑IL-10. (Syn): ↓IL-1β, ↓TNFα, ↑IL-10, ↓CD86, ↓Iba1, and ↑BDNF (Syn and EPEA): ↑IL-4, ↓MHC-II, ↑CD206, ↑Arg1, ↓GFAP, ↓S100β, and ↑LTP.	[87]
DPA	BV-2, SH-SY5Y cells	BV-2 cells; DPA (0, 12.5, 25, 50, or 100 µM) for 24 h + LPS (100 ng/mL) for 24 h; supernatant → SH-SY5Y cells	↑Cell viability, ↓NO, ↓Iba-1, ↓CD11b, ↑Arg1, ↑CD206, ↑IL-10, ↓IL-1β, ↓TNF-α, and ↓IL-1R1.	[88]

↓, decrease; ↑, increase; DHA, docosahexaenoic acid; LPS, lipopolysaccharide; IFN-γ, interferon-gamma; NO, nitric oxide; iNOS, inducible nitric oxide synthase; TNFα, tumor necrosis factor-alpha; Arg1, arginase-1; IL, interleukin; PGE2, prostaglandin E2; MAPK, mitogen-activated protein kinase; PPARγ, peroxisome proliferator-activated receptor gamma; NPC, neural stem progenitor cell; PUFAs, polyunsaturated fatty acids; LA, linoleic acid; AA, arachidonic acid; ALA, α-linoleic acid COX-2, cyclooxygenase-2; CD, cluster of differentiation; Erg-1, early growth response transcription factor-1; BDNF, brain-derived neurotropic factor; PhA, phytanic acid; ppm, parts per million; H_2_O_2_, hydrogen peroxide; LDH, lactate dehydrogenase; O2−, superoxide anion; GtPx, glutathione peroxidase; GtRd, glutathione reductase; SOD, superoxide dismutase; SD, Sprague Dawley; JEV, Japanese encephalitis virus; ROS, reactive oxygen species; NS1, nonstructural protein 1; IRF, interferon regulatory factor; P2X, P2X purinoceptor; Nrf2, nuclear factor erythroid 2-related factor 2; HO-1, heme oxygenase-1; NF-κB, nuclear factor kappa-light-chain-enhancer of activated B-cell; AP-1, activator protein 1; CREB, cAMP response element-binding protein; TLR, Toll-like receptor; MyD88, myeloid differentiation primary response 88; NLRP3, NLR family pyrin domain containing 3; ASC, apoptosis-associated speck-like protein containing a CARD; TAK1, transforming growth factor β-activated kinase-1; TBK1, TANK-binding kinase; ERK, extracellular signal-regulated kinase; JNK, c-Jun N-terminal kinase; Akt, serine/threonine-specific protein kinase; cPLA2, cytosolic phospholipase A2; JAK, Janus kinase; Tyk2, tyrosine kinase 2; Stat, signal transducers and activators of transcription; CCL, C-C motif ligand; SOCS3, suppressor of cytokine signaling 3; NGF, nerve growth factor; Syn, synaptamide; EPEA, N-eicosapentaenoylethanolamine; Iba1, ionized calcium-binding adaptor molecule-1; MHC-II, major histocompatibility complex-II; GFAP, glial fibrillary acidic protein; S100β, S100 calcium-binding protein β; LTP, long-term potentiation; DPA, docosapentaenoic acid.

### 3.2. Role of Fatty Acids in Microglial Polarization in Spinal Cord Injury (SCI)

SCI is the third leading cause of acquired disability worldwide. Limited pharmacological treatments are available for SCI. SCI is defined as mechanical damage that is followed by a complex molecular cascade leading to neuroinflammation [89,90].

An in vivo study using Wistar rats and primary microglial cultures demonstrated the role of DHA in promoting microglial polarization in the SCI model [91]. Treatment with DHA promoted microglial polarization by downregulating the expression of M1-specific markers, such as CD86, and upregulating the expression of the M2-specific marker CD206 and the activity of the antioxidant molecule superoxide dismutase 1 (SOD1) (Table 3). Another follow-up study [92] reported similar results. DHA administration enhanced open-field locomotor scores, improved neuronal survival, increased the expression of a microglial activation marker (ED1), decreased the number of Iba1-positive cells, reduced myelin phagocytosis, and increased miR-124 expression levels, in addition to increasing the expression levels of M1 (CD16/32) and M2 (Arg1) markers at the injury epicenter (Table 3). However, DHA downregulated the CD16/32 levels in LPS-induced or IFN-γ-induced bone marrow-derived macrophages from C57BL/6 mice. Additionally, DHA decreased the phagocytic activity and myelin phagocytosis in BV-2 and PC12 cells and upregulated miR-24 expression. This indicates the role of miR-24 in DHA-mediated effects (Table 3).

### 3.3. Role of Fatty Acids in Microglial Polarization in Alzheimer’s Disease (AD)

AD is progressive, irreversible, and the most prevalent ND. Additionally, AD is the most common cause of dementia, accounting for 50–70% of all dementia cases. AD is characterized by cognitive dysfunction, accumulation of amyloid-beta (Aβ) plaques in the cerebral cortex, and hyperphosphorylated tau protein-mediated neuronal damage that is initiated in the hippocampus and cortex region of the brain [93]. Aβ peptide and tau protein consistently accumulate in the frontal and/or parietal lobes and cause alterations in the frontal lobe that impact memory and error-driven learning in individuals who have a high risk of dementia: a novel manuscript provides an overview of the anatomical–functional interplay between the prefrontal cortex and heart-related dynamics in human emotional conditioning (learning) and proposes a theoretical model to conceptualize these psychophysiological processes, the neurovisceral integration model of fear (NVI-f), which can be impaired in the context of psychiatric disorders [94], while another recent study demonstrated, on a neurophysiological level, the role of the prefrontal cortex (PFC) in fear conditioning [95].

The most typical symptoms of this ND are neurodegeneration, which is characterized by gliosis and neuronal loss [96]. The development of a customized treatment strategy will heavily rely on peripheral biomarkers. Concerning this, Török and his colleagues paid close attention to how the kynurenine metabolic pathway (KP) works and how changes lead to NDs such as AD [97].

Our understanding of AD is also growing due to translational research based on “omics” tools, notably lipidomics, which is helping to identify early AD biomarkers. In this regard, the control of neurolipid-mediated signaling, which entails comprehension of the integration of structural, energetic, and signaling functions mediated by lipid molecules in AD patients, may aid in developing efficient therapy approaches for NDs [98].

In the early stages of AD, structural deformations in the frontal, parietal, and temporal cortices followed by the enlargement of the horn of the lateral ventricle and atrophy of the entorhinal cortex, amygdala, and hippocampus are characteristic findings in patients with AD. However, motor or sensory dysfunctions are not generally associated with the later stages of AD. The deposition of Aβ plaques is mainly responsible for activating microglia and astrocytes to release cytokines that further result in inflammation [96]. Neuroinflammatory responses have been observed in both sporadic and familial AD cases, as well as in AD transgenic models [99]. Additionally, the in vivo Aβ-induced AD model exhibits microglial activation and inflammatory responses, which are elicited by the binding of Aβ to innate immune receptors of microglia [100,101,102]. During the early stages of AD, microglial cells are activated to exert neuroprotective effects by reducing Aβ deposition [103] and mitigating tau hyperphosphorylation [104]. However, the later stages of AD are associated with aberrant activation of microglial cells, which subsequently promote inflammation and contribute to AD progression [105,106].

According to a recent study by Battaglia et al. [107], the cognitive dysfunctions seen in patients with AD/MCI may eventually result from age-related impairments in the capacity to process contextual information and in the regulation of responses to threats. These cognitive changes are determined by structural and physiological changes in the prefrontal cortex and medial temporal lobe.

DHA is an essential molecule in neurodevelopmental processes, and its function as an indirect antioxidant in the brain and its alterations during healthy and pathological aging, as well as in AD, were reviewed by Dáz et al. [108].

Hjorth et al. examined the role of ω-3 fatty acids in AD by focusing on their effects on microglial polarization [109]. CHME3 cells were treated with different concentrations of DHA and EPA, followed by treatment with Aβ42 for different durations. The in vitro data revealed that DHA and EPA promoted microglial polarization from the M1 phenotype to the M2 phenotype by downregulating the expression of M1-specific markers (CD40 and CD86) and upregulating the expression of an M2-specific marker (CD206) (Table 3). Neuroinflammation resulting from altered phagocytosis of Aβ42 is a key feature associated with the progression of AD. DHA and EPA can induce Aβ42 phagocytosis [110]. BDNF, which is reported to play an essential role in tau-related neurodegeneration in AD [111], is downregulated in the brain of patients with AD [112]. This study was the first to report the influence of DHA and EPA on the microglial production of BDNF. Pretreatment with DHA and EPA [66] before LPS stimulation resulted in the downregulation of anti-inflammatory cytokines, such as TNF-α and IL-6, in MG6 and BV-2 cells (Table 3).

Moreover, the protein deacetylase sirtuin1 (SIRT1) is reported to be involved in suppressing the inflammatory activity of classically activated microglia [113], as well as in microglia-mediated Aβ-induced neurotoxicity [114]. This was the first study to show the effects of EPA and DHA on the SIRT1 pathway. EPA and DHA upregulated the levels of nicotinamide phosphoribosyl transferase (NAMPT), SIRT1, and nicotinamide adenine dinucleotide (NAD+), which suppress TNF-α production. Additionally, EPA and DHA promoted SIRT1-dependent autophagy, which was assessed by examining the LC3-II/ LC3-I ratio (an indicator of autophagy), and inhibited NF-κB activation.

A recent study revealed similar immunomodulatory effects of EPA in the Aβ42-induced AD C57BL/6 model [115]. Administration of ethyl (E)-EPA for 42 days alleviated spatial learning and memory deficits and downregulated M1-specific chemokine C-C motif ligand (CCL) 2, which is reported to play an essential role in glial activation and tau pathology [116], and upregulated M2-specific Arg1 (Table 3). Additionally, ethyl (E)-EPA inhibited the release of pro-inflammatory cytokines and apoptotic neural death. BDNF and NGF, which are known to be involved in neuronal repair [117,118], were also upregulated upon EPA administration (Table 3).

**Table 3:** In vitro and in vivo studies on fatty acid-mediated microglial polarization in SCI and AD.

Model	Compound	Cell/Animal	Treatment	Findings	Ref.
SCI	DHA	Wistar rats PMCs	DHA (45 mg/kg bodyweight), 3 weeks post-SCI. DHA (1 μM) for 2 or 4 days.	↑Activation and proliferation of microglial cells, ↓CD86, and ↑CD163. ↑SOD and ↑microglial cell proliferation.	[91]
	DHA	SD rats C57BL6 mice BMDMs (C57BL/6 mice) PC12 cells BV-2 cells PMCs (Wistar rats)	DHA (500 nmol/kg bodyweight), 30 min post-SCI, 7 and 28 days, 35 days for C57BL/6 mice. LPS (100 ng/mL) or IFN-γ (20 ng/ mL) or IL-4 (20 ng/mL) for 30 min + DHA (1 or 3 μM) for 24 h. UV-stressed PC12 + DHA-treated BV-2 cells (DHA; 3 μM for 24 h) DHA (1 and 3 μM) for 24 h	↑Locomotor recovery, ↑neuronal survival, ↑ED1, ↑CD16/32, ↑Arg1, ↑miR-124, and ↓myelin phagocytosis. ↓M1 (CD16/32) ↓Phagocytic activity of microglia, ↓myelin phagocytosis, and ↑miR-124 ↑miR-124	[92]
AD	DHA and EPA	CHME3 cells	DHA or EPA (0.05, 0.1, 0.5, or 1 μM) + Aβ42 (1 μg/mL) for 2, 6, or 24 h.	↑Aβ42-Phagocytosis, ↑BDNF, ↓TNF-α, ↓CD40, ↓CD86, and ↑CD206.	[109]
	DHA and EPA	MG6 cells BV-2 cells	DHA or EPA (200 μM each), DHA + EPA (100 μM) for 30 min + LPS (100 ng/mL) for 24 h.	↓TNF-α, ↓IL-6, ↑SIRT1, ↓NF-κB, ↑NAMPT, ↑NAD^+^, and ↑autophagy.	[66]
	EPA	C57BL/6 mice	Ethyl (E)-EPA (0.8%; 8–10 g per mouse/day) for 42 days. On day 28, treat with 2 μg Aβ.	↑Spatial learning and memory, ↓CCL-2, ↑Arg1, ↓IL-1β, ↓TNF-α, ↓IL-6, ↑BDNF, ↑NGF, and ↓neural death.	[115]

↓, decrease; ↑, increase; SCI, spinal cord injury, DHA, docosahexaenoic acid; PMCs, primary microglial cells; CD, cluster of differentiation; SD, Sprague Dawley; ED1, a marker of activated microglia and monocyte-derived macrophages; Arg1, arginase 1; BMDMs, bone marrow-derived macrophages; LPS, lipopolysaccharide; IFN-γ, interferon-gamma; IL, interleukin; AD, Alzheimer’s disease; EPA, eicosapentaenoic acid; Aβ42, beta-amyloid (1–42); BDNF, brain-derived neurotrophic factor; SIRT1, sirtuin1; NF-κB, nuclear factor kappa-light-chain-enhancer of activated B cells; NAMPT, nicotinamide phosphoribosyl transferase; NAD, nicotinamide adenine dinucleotide; CCL, C-C motif ligand; TNF-α, tumor necrosis factor-α; NGF, nerve growth factor.

### 3.4. Role of Fatty Acids in Microglial Polarization in MS

MS, a CNS disease, affects approximately 2.8 million individuals worldwide (35.9 per 100,000 population) [119]. The characteristic features of MS include autoimmunity, microglial activation, demyelination of neurons, monocyte infiltration, and neuronal death [120,121]. The most common symptoms of MS involve dysfunction of psychobehavioral disturbances, such as paresthesia, gait difficulties, blurred vision, vertigo, dizziness, sexual problems, constipation, incontinence, spasticity pain, cognitive impairment, and depression. The neural lesion of MS is represented by numerous glial scars, called plaques, which develop in the white matter and spinal cord [122]. Activated mononuclear cells such as microglia, macrophages, and lymphocytes from active plaques cause damage to myelin and oligodendrocytes, which degrade into myelin lipids, proteins, and debris. These plaques result in demyelinated axons traversing glial scar tissue, forming inactive plaques. Shadow plaques are formed by partially remyelinated plaques formed by oligodendrocytes. Others are smoldering plaques that expand to the peripheral region but are inactive at the center [123,124].

MS is a typical example of inflammatory demyelination. Demyelination of particular structures and imbalance due to the interaction of a dysregulated immune system and specific lymphocyte receptors can lead to negative impacts such as neuropsychiatric, autonomic, cognitive, and sleep complications in MS. In this regard, research in healthy people has demonstrated that the modulation of autonomic nervous system responses is critical for behavioral regulation, showing how this function is compromised in MS patients [96,125,126,127].

Microglial cells have detrimental and beneficial roles in demyelination (by presenting antigens and secreting pro-inflammatory molecules that can damage the myelin sheath and/or oligodendrocytes) and remyelination of neurons (by promoting the expression of anti-inflammatory molecules, phagocytosis of debris, and repair of tissues) [128].

A study investigated the role of ω-3 PUFAs in microglial polarization in primary microglial cultures and a cuprizone (CPZ)-induced MS model [129]. Primary microglial cells were pretreated with DHA or EPA and stimulated with LPS, which resulted in the downregulation of NO, TNF-α, IL-6, and M1-specific markers, such as CCL5, CD14, CCL2, IL-23a, CCL4, IL-1α, and iNOS, and the upregulation of myelin phagocytosis and M2-specific markers (CCL22, CCL17, Arg1, IL-5, chitinase (Chi) 311, Gata3, CD206, CCL11, CCR2, transforming growth factor (TGF) β, and CCL1) (Table 4). The administration of ω-3 PUFAs to the CPZ-induced MS model (model for toxic demyelination) for 5 weeks exerted neuroprotective effects by alleviating demyelination and neurological deficits, downregulating M1-specific markers (CD16 and iNOS), and upregulating M2-specific markers (CD206, chitinase-like protein (Ym-1/2), and Arg1). Monte et al. also confirmed the immunomodulatory effects of fatty acid dietary supplements in an in vivo experimental autoimmune encephalomyelitis (EAE) model of MS [130]. EAE mice [131] were administered a neuro (n)FAG suspension (which contains a different mix of FAs in different proportions, plus lycopene, and has been developed to specifically treat inflammatory events that damage retinal ganglion cells and their axons) for 16 days, which delayed the onset of EAE and downregulated the expression of pro-inflammatory cytokines and M1-specific markers (Table 4). Locri et al. [132] reported a microglial shift in the EAE model upon dietary supplementation of saturated and unsaturated fatty acids. This microglial shift alleviated optic nerve damage and prevented the reduction in the photopic negative response (PhNR) amplitude, a sensitive marker of inner retinal function [133]. STAT3 is considered a major anti-inflammatory mediator that represses pro-inflammatory cytokines [134,135]. In contrast, NF-κB modulates the expression of various pro-inflammatory cytokines in response to various physiological and environmental stimuli [136]. Previous studies have also demonstrated the correlation between decreased STAT3 activity and increased EAE severity, which could be ameliorated by NF-κB inactivation [137]. In this study, fatty acid supplementation upregulated the phosphorylation of STAT3 but downregulated that of NF-κB (Table 4).

α-Linoleic acid (ALA) and valproic acid (VA) conjugates (1 and 2) prepared by Rossi et al. exerted neuroprotective effects in the MS model [138]. These conjugates were effective against an LPS-induced M1-specific marker (iNOS) in N9 microglial cells and were more efficient than ALA and VA alone or in combination. Oligodendrocyte precursor cell (OPC) proliferation and migration to the injury site in the CNS are crucial steps in remyelination in MS [139]. Oli-Neu cells treated with VPA, ALA, and their conjugates promoted OPC differentiation into oligodendrocytes (Table 4). Conjugate- and VPA-treated groups exhibited decreased oligodendrocyte transcription factor 2 (Olig2, a marker of OPC proliferation) expression. Additionally, both conjugate 1 and VPA significantly upregulated the expression of 2′,3′-cyclic-nucleotide 3′-phosphodiesterase (CNPase, a marker of differentiation). These results support the role of conjugates and VPA in OPC differentiation and suggest that they regulate myelination/remyelination. The effect exerted by conjugate 1 was not exerted by ALA alone or in combination with VPA. A recent study reported that ω-3 PUFAs improved motor and cognitive abilities and myelin sheet recovery, upregulated the expression of antioxidant enzymes, and promoted the microglial shift in the CPZ-induced MS model [140]. Histone deacetylase 3 (HDAC3) is reported to regulate inflammation [141,142]. Previous studies have reported that HDAC3 inhibitors or HDAC3-deficient macrophages exert neuroprotective effects [143,144]. Supplementation with ω-3 PUFAs significantly downregulated CPZ-induced HDAC3 and NF-κB expression. Additionally, ω-3 PUFA supplementation downregulated the expression of p-STAT3, which is involved in MS progression [145,146] (Table 4).

### 3.5. Role of Fatty Acids in Microglial Polarization in Cerebral Ischemia

Ischemic stroke, which is one of the leading causes of death and disability in developing countries [147], is caused by an artery blockage that restricts oxygen-rich blood from reaching the brain, resulting in brain tissue injury. Cerebral ischemia-associated blockage is often caused by blood clots. Neuroinflammation is considered one of the major causes of cerebral ischemia-induced brain damage and is initiated in response to ischemic stroke. Over-activation of microglial cells releases various pro-inflammatory cytokines that exacerbate cerebral ischemia–reperfusion (I/R) injury [148,149]. There are many different cytokines reported as biomarkers for cerebral ischemia, such as IL-1β, IL-6, IL-8, IL-12, IL-15, IL-16, IL-20, IL-18, IL-23/IL-17, and so on, which play a pro-inflammatory role after ischemic stroke; ILs that have anti-inflammatory effects on ischemic stroke include IL-2, IL-4, IL-10, IL-13, IL-19, IL-33, and so on, as reported recently [150]. In 2019, ischemic stroke affected approximately 77.2 million individuals [151].

Zended et al. performed in vivo and in vitro experiments using Wistar rats, primary astroglial and neuronal cultures, and BV-2 microglial cells and demonstrated the effect of DHA and EPA on cerebral ischemia [152]. DHA and EPA alleviated post-stroke ischemic damage and behavioral deficits in Wistar rats and downregulated the expression of hypoxia-inducible factor 1 alpha (HIF1α), which is involved in destabilizing BBB integrity [153,154]. Tau and growth-associated protein-43, which are marker genes for axonal integrity and dendritic plasticity, respectively, were downregulated after transient middle cerebral artery occlusion (tMCAO). However, ω-3 PUFA supplementation mitigated the tMCAO-induced downregulation of these marker genes (Table 4) and mRNA levels of inflammatory cytokines and the inflammasome (NLRP3). DHA and EPA significantly downregulated the inflammatory markers TNF-α, iNOS, CCL5, and COX-2 in hypoxic-primary neuronal cell cultures and BV-2 microglia. Additionally, DHA and EPA shifted the Bax (Bcl-2 associated X, apoptosis regulator)/Bcl-2 (B-cell lymphoma 2) protein ratio by upregulating Bcl-2 expression and downregulating Bax expression, which protected against apoptosis [155]. Jiang et al. reported that DHA alone or in combination with fish oil (FO) alleviated motor deficits and brain tissue loss in a transient focal cerebral ischemia C57BL/6J mouse model [156]. Myelin basic protein (MBP) and SMI-32 are markers of myelination and demyelination, respectively. DHA and FO potentiated the MCAO-induced downregulation of MBP and upregulation of SMI-32 (Table 4). The regeneration of myelinating oligodendrocytes is crucial for remyelination, neurological recovery, and white matter restoration [157,158]. Oligodendrogenesis was significantly upregulated in the DHA- and FO-treated groups, as evidenced by an increased number of mature oligodendrocytes expressing markers of oligodendrocyte maturity, including 5-bromo-2′-deoxyuridine and APC [159]. Additionally, DHA decreased the numbers of M1 (CD16/32+/Iba1+) cells in the peri-infarct CC, cortex, and striatum and increased the M2 (CD206+/Iba1+) cell numbers in the cortex and striatum. The combination of DHA and FO did not affect the number of CD16/32+/Iba1+ cells but increased the number of CD206+/Iba1+ cells in the CC, cortex, and striatum.

Cai et al. demonstrated the role of DHA in alleviating acute ischemic brain injury and modulating M1/M2 phenotypes [160]. Post-stroke DHA administration efficiently alleviated neurological deficits and brain injury on day 3 post-tMCAO and inhibited the infiltration of macrophages, neutrophils, T lymphocytes, and B lymphocytes. DHA administration increased the percentage of M2 phagocytes without affecting the percentage of M1 cells in the ischemic brain. An increase in anti-inflammatory markers and a gradual decrease in pro-inflammatory markers and chemokines were considered additive effects. DHA did not exert therapeutic effects in macrophage-depleted animals, which indicated that the modulatory effects were mediated through macrophages (Table 4). The results of in vitro experiments were consistent with those of in vivo experiments. DHA mitigated the LPS-induced downregulation of mRNA expression of M1-specific markers and upregulation of M2-specific markers. Another supporting study demonstrated that EPA and DHA emulsion treatment promoted the microglial shift in the tMCAO-induced model [161]. Additionally, EPA and DHA emulsion treatment upregulated the levels of hematopoietic tissue surface (HPS), downregulated M1-specific marker (iNOS2) expression, and increased M2-specific marker (Arg1) expression. The same group published another study using FO (ω-3 PUFAs are the major components of dietary FO). Preconditioning with FO promoted the polarization of splenic macrophages toward the M2 phenotype in experimentally induced transient cerebral ischemia [162]. FO mitigated tMCAO-induced splenic morphological changes in Wistar rats administered a phospholipid emulsion for 21 days, followed by the induction of tMCAO. Additionally, FO mitigated the tMCAO-induced splenic extra-medullary granulocytic lineage and changes in the M1/M2 profile. In particular, FO mitigated tMCAO-induced iNOS2 upregulation and Arg1 downregulation (Table 4).

Recently, Zhang et al. [163] examined the presence of NALP3, M1, and M2-specific cells in human and I/R mouse brain tissues. M1 cells exhibited the expression of iNOS, an early pro-inflammatory marker. Meanwhile, M2 cells exhibited the expression of Ym-1, a late anti-inflammatory marker (2 days after I/R). DHA significantly decreased the number of NALP3+ cells and alleviated brain injury in MCAO C57/BL6 mice (Table 4). Peritoneal macrophages treated with IFN-γ and LPS (to stimulate M1 macrophages) (iNOS- and CD11b/CD80-positive cells) or IL-4 (to stimulate M2 macrophages) (CD206- and CD11b/CD206-positive cells), subjected to oxygen/glucose deprivation for 1 h, and treated with HMBG1 exhibited enhanced activation of NALP3 and IL-1β, which was mitigated upon treatment with DHA. Additionally, DHA decreased M1 polarization, as evidenced by the downregulated expression of IL-1β. Furthermore, DHA decreased the number of M1 macrophages (iNOS-TRITC/CD11b-FITC) but increased the number of M2 macrophages (Arg1-TRITC/CD11b-FITC) in the penumbra of ischemic murine brain tissue collected 2 days after cerebral I/R injury. The expression of M1 macrophage-associated genes (iNOS/IL-1β/IL-23) was downregulated, whereas that of M2 macrophage-associated genes (Arg1/Ym-1/IL-10) was upregulated (Table 4).

**Table 4:** In vitro and in vivo studies on fatty acid-mediated microglial polarization in MS and cerebral ischemia.

Model	Compound	Cell/Animal Type	Treatment	Findings	Ref.
MS	DHA and EPA	PMCs C57/BL6 mice	DHA or EPA (5–80 mM) for 24 h + LPS (2.5 ng/mL) for 24 h or DHA or EPA (20 mM) for 24 h + myelin (1, 5, or 10 mg/mL) w/o IFN-γ (5 ng/mL) for 24 h. Cuprizone (0.2%) with low ω-3 PUFA (0.3%) or with high ω-3 PUFA (DHA 1 EPA, 15 g/kg bodyweight) for 5 weeks.	↓NO, ↓TNF-α, ↑myelin phagocytosis, ↓IL-6, ↓CCL5, ↓TNF-α, ↓CD14, ↓CCL2, ↓IL-23a, ↓CCL4, ↓IL-1α, ↓iNOS, ↑CCL22, ↑CCL17, ↑Arg1, ↑IL-5, ↑Chi3l1, ↑Gata3, ↑CD206, ↑CCL11, ↑CCR2, ↑TGFβ, and ↑CCL1. ↓Demyelination, ↓neurological deficits, ↓CD16, ↓iNOS, ↑CD206, ↑YM1/2, and ↑Arg1.	[129]
	nFAG	C57BL/6J mice	nFAG (10 mg/daily) on day 16 post-immunization with MOG.	Delays the onset of EAE, ↓TNF-α, ↓IL-1β, ↓IL-6, ↓IL-8, ↓ICAM-1, ↓GFAP, ↓iNOS, ↓RGC degeneration, and ↓RGC loss.	[130]
	SFAs and USFAs	C57BL/6J mice	9 MOG-treated mice (20% SFAs + 17.5% USFAs) for 16 days.	↓CXCL-10, ↓CXCL-11, ↓IL-12, ↓IL-23, ↑CCL-2, ↑CCL-22, ↑CD163, ↑Arg1, ↑IL-10, ↓NF-κB, ↓optic nerve damage, and ↑PhNR amplitude	[132]
	ALA and VA	N9 microglial cells Oli-Neu cells CGNs	LPS (100 ng/mL) + VPA or ALA or diamide 1 or ethanolamide 2 (0.5, 1.5, or 10 μM) and VPA + ALA (1:1) Each compound (1–10 μM) for 24 h. Each compound was pretreated (5, 10, or 25 μM) for 6 h + co-treatment with glutamate (100 μM)/glycine (10 μM) + 24 h.	↓iNOS ↑OPC differentiation, ↓Olig2, and ↑CNPase ↑Neuroprotection	[138]
	KO diet (rich in ω-3 PUFAs)	C57BL/6 mice	KO diet containing 0.2% CPZ for 5 weeks.	↓Motor abnormalities and cognitive deficit, ↑GSH-Px, ↑SOD, ↑GSH, ↓MDA, ↑myelin sheet recovery, ↓NG2^+^ OPCs, ↓CD68+ cells, ↓Iba1+ cells, ↓CD16, ↓iNOS, ↑Arg1, ↑CD206, ↓HDAC3, ↓p-STAT3, and ↓NF-κB.	[140]
Cerebral Ischemia	ω-3 PUFAs	Wistar Rats, primary astroglial and neuronal cultures, BV-2 microglia	MCAO + DHA (140 mg/kg bodyweight/day), EPA (220 mg/kg bodyweight/day) for 24 h. Hypoxia for 1 h (neuronal cells) or 3 h (astroglia, BV-2 cells) w/o emulsion.	↓Ischemic damage, ↑behavioral performance, ↑GAP-43, ↑Tau mRNA, ↓HIF1a, ↓IL-1β, ↓TNF-α, ↓NLRP3, ↓Arg1. ↓TNF-α, ↓iNOS, ↓CCL5, ↓COX-2, ↓Bax, and ↑Bcl-2	[152]
	DHA and FO	C57BL/6J mice	MCAO for 2 h + DHA (10 mg/kg bodyweight/day) for 14 days w/o FO supplementation and 5 days post-MCAO	↑Long-term histological and functional outcomes, ↓white matter injury, ↑BrdU, ↑APC, ↑MBP, ↓SMI-32, ↓CD16/32^+^/Iba1^+^ cells, and ↑CD206^+^/Iba1^+^ cells	[156]
	DHA	C57BL/6 mice	tMCAO for 1 h + DHA (10 mg/kg bodyweight) for 3 days.	↓Brain infarct, ↓neurological deficit, ↓infiltration (macrophages, neutrophils, T and B lymphocytes), ↑CD206^+^ CD16^-^, ↑CD206^+^Iba1^+^, ↑CD206^+^CD16^−^, ↑IL-10, ↑Arg1, ↑TGFβ, ↓CCL1, ↓CCL2, ↓CCL3, ↓CCL17, ↓CXCL1, ↓CXCL2, ↓CXCL10, ↓CXCL12, ↓CXCL13, ↓C5/5a, ↓IL-1α, ↓IL-1rα, ↓IL-27, ↓IFNγ, ↓TNFα, ↓GCSF, ↓TIMP1, and ↓TREM1.	[160]
	FO	Wistar rats	Phospholipid emulsion; EPA (70 mg/kg bodyweight) and DHA (80 mg/kg bodyweight) daily for 21 days post-MCAO.	↑HPS, ↓iNOS, ↑Arg1, ↓granulopoiesis, and ↓myeloperoxidase positivity	[161]
	DHA	Human and I/R mouse brain tissue C57/BL6 mice Mouse peritoneal macrophages and brain microglia	Assessed for NALP3 and M1 and M2 cells. tMCAO + DHA (5 mg/kg bodyweight) after tMCAO. DHA (1 µmol/L) on days 2 and 10. After 24 h, treated with IFN-γ (100 U/mL), LPS (100 ng/mL), and IL-4 (100 U/mL) for 72 h.	↑NALP3+ cells on day 4, but decreased on day 6, ↑iNOS, and ↑Ym-1 ↓I/R injury, ↓iNOS, ↓IL-1β, ↓IL-23 ↑Arg1, ↑Ym-1, ↑IL-10, and ↓NALP3 ↓NALP3 and ↓IL-1β	[162]

↓, decrease; ↑, increase; MS, multiple sclerosis; DHA, docosahexaenoic acid; EPA, eicosapentaenoic acid; PMCs, primary microglial cells; LPS, lipopolysaccharide; IFN-γ, interferon-gamma; PUFAs, polyunsaturated fatty acids; NO, nitric oxide; TNF-α, tumor necrosis factor-α; IL, interleukin; CCL, C-C motif ligand; iNOS, inducible nitric oxide synthase; Arg1, arginase 1; Chi3l1, chitinase 3 like-1; GATA3, GATA binding protein 3; CCR2, C-C chemokine receptor type 2; TGFβ, transforming growth factor-beta; CCL1, C-C motif chemokine ligand 1; Ym1/2, chitinase-like protein; nFAG, neuro-FAG; MOG, myelin oligodendrocyte glycoprotein; EAE, experimental autoimmune encephalomyelitis; ICAM-1, intercellular adhesion molecule-1; GFAP, glial fibrillary acidic protein; RGC, retinal ganglion cell; SFAs, saturated fatty acids; USFAs, unsaturated fatty acids; CXCL, C-X-C motif ligand; NF-κB, nuclear factor kappa-light-chain-enhancer of activated B-cell; PhNR, photopic negative response; ALA, α-linoleic acid; VA, valproic acid; CGNs, cerebellar granule neurons; OPCs, oligodendrocyte precursor cells; Olig2, oligodendrocyte transcription factor; CNPase, 2′,3′-cyclic-nucleotide 3′-phosphodiesterase; KO, krill oil; CPZ, cuprizone; GSH-Px, glutathione peroxidase; SOD, superoxide dismutase; GSH, glutathione; MDA, malondialdehyde; NG2, neuron-glial antigen 2; Iba1, ionized calcium-binding adaptor molecule-1; HDAC, histone deacetylase; STAT, signal transducer and activator of transcription; CI, cerebral ischemia; MCAO, middle cerebral artery occlusion; HIF1a, hypoxia-inducible factor 1 alpha; COX-2, cyclooxygenase-2; Bax, Bcl-2 associated X; Bcl-2, B-cell lymphoma 2; FO, fish oil; BrdU, 5-bromo-2′-deoxyuridine; APC, adenomatous polyposis coli; MBP, myelin basic protein; SMI-32, nonphosphorylated neurofilaments; tMCAO, transient middle cerebral artery occlusion; TIMP1, tissue inhibitor of metalloproteinase-1; TREM1; triggering receptor expressed on myeloid cells-1; HPS, hematopoietic tissue surface; NALP3, NACHT-LRR-PYD-containing protein 3 inflammasome; Ym-1, chitinase-like protein.

### 3.6. Role of Fatty Acids in Microglial Polarization in Traumatic Brain Injury (TBI)

TBI is a serious head injury that disrupts the physiological functioning of the brain and is considered a critical risk factor for NDs, such as AD [164,165,166], PD [167,168], MS [169], and ALS [170]. A bump, jolt, or blow to the head can result in TBI [171].

A study published in 2015 demonstrated that DHA exerted anti-inflammatory effects and altered microglial activation in the TBI Sprague Dawley (SD) rat model [172]. Animals that received DHA after the onset of TBI for 21 days exhibited a moderate decrease in the number of CD16/CD32+/Iba1+ cells on days 3 and 7 post-TBI. Additionally, DHA treatment mitigated the TBI-induced impaired branching of Iba1+ cells. DHA treatment induced a morphological shift away from an amoeboid-like shape and toward the classical branched morphology with cellular extensions protruding from the cell body, which were hypertrophic, resulting in a reduction in the number of endpoints and a decrease in the summed average length of microglial or macrophage processes on day 3 post-TBI. Administration of DHA post-TBI significantly increased the number of CD206+/Iba1+ cells between days 3 and 21. Endoplasmic reticulum (ER) stress-associated inflammation is a crucial trigger for several inflammatory pathways [173]. The ER stress marker C/-EBP homologous protein (CHOP) was upregulated in neurons, which were surrounded by Iba1+ microglial cells on day 3 post-TBI. However, treatment with DHA post-injury decreased the number of CHOP+ neurons and the association between neurons and Iba1+ microglial cells. DHA treatment increased the number of vacuoles associated with phagocytic activity in microglia and downregulated the expression of the lysosomal marker lysosomal-associated membrane protein 1 (LAMP)-1 post-TBI. Additionally, DHA mitigated TBI-induced NF-κB nuclear translocation, TNF-α upregulation, and neuronal degeneration in the brain (Table 5). ALA exerted preventive effects on a TBI mouse model [174]. An adequate amount of ALA induced increased production of DHA in the brain, which corresponded to the low production of ω-6 fatty acids (arachidonic acid, docosatetraenoic acid, and DPA) in TBI mice. Animals exhibiting upregulated levels of DHA were immune to neuroinflammation after TBI and exhibited downregulated expression of pro-inflammatory cytokines. Additionally, DHA-deficient mice exhibited increased levels of GFAP, which indicates increased astrocyte activation. Only CD206 was significantly upregulated in the low-ALA diet group (Table 5). Mice with increased levels of DHA in the brains exhibited improved motor function. Although the role of ALA in modulating the microglial phenotype is unclear, it modulated CD206 expression and exhibited anti-inflammatory and neuroprotective properties. This indicates that ALA is an important dietary fatty acid.

Chen et al. (2018) reported the effects of ω-3 PUFAs in TBI-induced neuroinflammation in SD rats [83]. Administration of ω-3 PUFAs improved neurological functions and brain water content, decreased neuronal apoptosis, downregulated an M1-specific marker (CD16+) and pro-inflammatory factors (IL-1β, TNF-α, and IL-6), and upregulated an M2-specific marker (CD206+) and anti-inflammatory cytokine (IL-10). High mobility group box protein 1 (HMGB1), a ubiquitous nuclear protein released by glia and neurons, is a potential target for TBI-induced neuroinflammation [175]. Additionally, the NF-κB pathway is reported to be associated with HMGB1 expression [176]. Supplementation with ω-3 PUFAs downregulated HMGB1 expression and NF-κB translocation and binding activity. SIRT1 is an NAD+-dependent histone deacetylase. Post-translational modifications, such as acetylation, are critical factors for HMGB1 transcription and its extracellular secretion [177]. In particular, deacetylation is known to inhibit HMGB1 transcription [178]. ω-3 PUFA supplementation increased SIRT1 expression and deacetylase activity. This study revealed a novel counter-regulatory relationship between the attenuation of TBI-mediated HMGB1/NF-kB p65 pathway activity and the regulation of SIRT1 overexpression (Table 5).

Schober et al. also demonstrated the short-term therapeutic effects of DHA in the TBI SD rat model [179]. DHA downregulated the nitrate/nitrite levels, mitigated controlled cortical impact-induced microgliosis, decreased the expression of CD68 and MHC-II, and upregulated the expression of CD206 (days 3 and 7 post-injury) (Table 5).

### 3.7. Role of Fatty Acids in Microglial Polarization in Depression

Depression is a psychological disorder that affects approximately 322 million individuals worldwide (approximately 4.4% of the world population). This pathological condition impairs the ability to perform everyday tasks. Chronic depression could lead to an increased risk of poor prognosis among patients with chronic diseases [180,181]. Although depression and NDs are considered to be different clinical conditions, their co-occurrence has been reported. The incidence of depression among patients with AD and PD is 90% and 50%, respectively [181]. Neuroinflammation is a common link between depression and NDs.

Heterozygous transgenic Fat-1 mice (the Fat-1 gene converts ω-6 to ω-3 PUFAs endogenously) were administered soybean oil and injected with LPS. ω-3 Fatty acids exerted modulatory effects on depression-like behavior [182]. The expression levels of M1-specific inflammatory cytokines and markers (IL-1β, TNF-α, and IL-17) and CD11b were downregulated, whereas those of M2-specific cytokines (IL-4, IL-10, and TGF1β) and BDNF were upregulated in ω-3 fatty acid-treated animals. Moreover, the levels of oxidative stress-related markers (iNOS and NO) in Fat-1-expressing LPS-treated mice were downregulated when compared with those in LPS-treated mice (Table 5). Recently, Wu et al. demonstrated the potential of FO containing PUFAs in alleviating depression-like behavior [183]. The administration of FO comprising EPA and DHA before ovariectomy (OVX) exerted anti-anxiety effects, decreased the number of apoptotic cells, and downregulated the expression of the microglial activation marker Iba-1. Additionally, FO promoted a microglial shift, as evidenced by the downregulation of M1-specific cytokines and markers (IL-1β, TNF-α, IL-6, CD86, and iNOS) and the upregulation of M2-specific cytokines and markers (IL-10, IL-4, CD206, and Arg1). This further indicates the role of ω-3 fatty acids in promoting microglial polarization. Furthermore, FO downregulated NF-κB signaling and upregulated IB kinase (IκB) activity (Table 5).

**Table 5:** In vitro and in vivo studies on fatty acid-mediated microglial polarization in TBI and depression.

Model	Compound	Cell/Animal Type	Treatment	Findings	Ref.
TBI	DHA	SD rats	TBI for 5 min + DHA (16 mg/kg bodyweight/daily in DMSO) for 3, 7, or 21 days.	↓CD16/32^+^, ↑CD206^+^, ↓ER stress, ↓NF-κB, ↓CHOP^+^ neurons, ↓LAMP-1, and ↓TNF-α.	[172]
	ALA	Pregnant C57BL6/N mice	Flaxseed oil (3.1% ALA) for 4 months + TBI.	↑Brain DHA, ↓DPA/DHA, ↓IL-1β, ↓TNFα, ↓IL-6, ↓CCL2, ↑CD206, ↓GFAP, and ↓motor and cognitive abilities	[174]
	DHA	SD rats	CCI for 30 min + DHA (100–150 mg/kg bodyweight/day)	↓Nitrate/nitrite, ↓microgliosis, ↓CD68, ↓MHC-II, ↑CD206, ↓cell number, ↓CD86, ↓CD32, ↓IL-1 β, ↓TNF-α, ↓IL-10, ↓TGFβ, and ↑novel object recognition	[83]
	ω-3 PUFA	SD rats	TBI for 30 min + ω-3 PUFA (2 mL/kg bodyweight/day) for 7 days.	↑Neurological functions, ↓brain water content, ↓neuronal apoptosis, ↓CD16^+^, ↑CD206^+^, ↓IL-1β, ↓TNF-α, ↓IL-6, ↑IL-10, ↓HMGB1/NF-κB, and ↑SIRT1	[179]
Depression	Soybean oil	Heterozygous transgenic Fat-1 mice	Soybean oil (10%) + 3 µg/µL LPS for 24 h.	↓CD11b, ↓IL-1β, ↓TNF-α, ↓IL-17, ↑IL-4, ↑IL-10, ↑TGF-β1, ↑BDNF, ↑TrkB, ↓p75, ↓NO, and ↓iNOS.	[182]
	DHA and EPA	SD rats	OVX surgery +1.5 g refined fish oil/kg bodyweight (approximate EPA and DHA contents were 340 and 240 mg/g, respectively) for 10 weeks.	↑Anti-anxiety, ↓apoptotic cells, ↓Iba-1, ↓IL-1β, ↓IL-6, ↓TNF-α, ↓CD86, ↓iNOS, ↑IL-10, ↑IL-4, ↑CD206, ↑Arg1, ↓NF-κB, and ↑IκB.	[183]

↓, decrease; ↑, increase; TBI, traumatic brain injury; DHA, docosahexaenoic acid; SD, Sprague Dawley; CD, cluster of differentiation; ER, endoplasmic reticulum; DMSO, dimethyl sulfoxide; NF-κB, nuclear factor kappa-light-chain-enhancer of activated B-cell; CHOP, C/EBP homologous protein; LAMP-1, lysosomal-associated membrane protein 1; TNF-α, tumor necrosis factor-alpha; ALA, α-linoleic acid; DPA, docosapentaenoic acid; IL, interleukin; CCL2, C-C motif ligand; CD, cluster of differentiation; GFAP, glial fibrillary acidic protein; CCI, controlled cortical impact; MHC-II, major histocompatibility complex-II; HMGB1, high mobility group box protein 1; SIRT1, sirtuin 1; LPS, lipopolysaccharide; TGF-β1, transforming growth factor-beta 1; BDNF, brain-derived neurotrophic factor; TrkB, tyrosine receptor kinase B; NO, nitric oxide; iNOS, inducible nitric oxide synthase; OVX, ovariectomy; IκB, IkappaB kinase.

## 4. Conclusions

In this review, we summarize the role of various fatty acids, particularly PUFAs, in tuning microglial polarization and in neuroprotection using cell and animal models. It is well known that an inflammatory microglial state is common in various neurodegenerative disorders, particularly Alzheimer’s disease. As per the gathered information, fatty acids, DHA, and EPA were shown to activate Aβ 42 phagocytosis, boosting the activation and proliferation of microglial cells, downregulating the M1 marker (CD86), and upregulating the M2 marker (CD163). It was shown that DHA and EPA increased the expression of SIRT1, a factor recognized for reducing inflammation that activates the M1 state and causes neurotoxicity. The review also clarifies how fatty acids increase the expression of BDNF, which is crucial for tau-related neurodegeneration in AD patients. In MS, DHA and EPA were observed to upregulate M2-specific markers (TGFβ, Arg1, IL-5, and CD206) while downregulating inflammatory cytokines (TNF-α and IL-6) and M1-specific markers (CCL5, CD14, IL-1, and iNOS). Notably, in animal models of cerebral ischemia, DHA and EPA suppressed the production of hypoxia-inducible factor 1 alpha (HIF1), a recognized agent that compromises BBB integrity.

Additionally, it was found that both of these fatty acids play a significant role in up- and downregulating the expression of Bcl-2 and Bax, confirming their anti-apoptotic effects. We also reviewed the protective effects of DHA, ALA, and EPA on traumatic brain injury and depression by upregulating and downregulating certain markers and cytokines (Table 5). Overall, the study discovered that PUFAs primarily significantly increased the percentage of the anti-inflammatory phenotype in microglia and blocked a number of neuroinflammation-related signaling pathways, including NF-B, MAPK, and TLR. Additionally, fatty acids prevented neuronal apoptosis, which is essential for treating NDs. Data on human research is, however, limited. More research is therefore required on unstudied fatty acids, their functions in microglial polarization, and their molecular implications for preventing neuroinflammation. Future studies are necessary to fully utilize fatty acids’ abilities to polarize microglia and provide neuroprotection against a variety of neurodegenerative diseases. These studies should be based on preclinical and clinical models.

## 5. Limitations and Future Directions

These findings show that fatty acids have direct and indirect effects on microglia polarization and neuroprotection. However, there are still a significant number of open issues. Extensive research is necessary to completely comprehend the status (M2a, M2b, M2c, and M2d) and functions of microglia. However, for therapy to be effective, M1 microglia must be inhibited and M2 microglia must be stimulated simultaneously [184]. Another issue is that it is still unclear whether microglia engagement and the associated inflammatory process follow a predictable pattern or whether environmental and inherited factors may play a role. To fully recreate the polarization process, it is crucial to thoroughly examine potential contributing elements, such as transcription factors, microRNA, and drug delivery. The ability to examine the connections of fatty acids or other targeted functional molecules is also restricted in animal models of only certain features of human neurodegenerative disorders. Furthermore, research into the connections between polarization and the recently discovered microglial classes PAM, DAM1, and DAM2 is still in its early stages.

Despite these limitations, we advise that future studies concentrate on fatty acids and how they affect the metabolic reprogramming of microglia, particularly mitochondrial energy metabolism. Another area for investigation is the function of the microbiome in microglial polarization [49] and the gastrointestinal–brain axis in NDs [97]. Future studies should concentrate on previously unstudied ND models and investigate the neuroprotective properties of fatty acids combined with other natural substances. Before experimental confirmation, fatty acids and other potential molecules that regulate microglial polarization can be found and characterized using bioinformatics methods and machine learning. Last but not least, how other risk genes affect microglia polarization in ND is still unknown.

## Figures and Tables

**Table 1:** Microglial phenotypes and their characteristics.

Pheno-Type	Sub-Type	Specific Cytokines	Chemokines	Specific Markers	Functions	Ref.
M1	-	IL-1β, IL-6, IL-12, IL-23, and TNF-α	CCL4, CCL5, CCL8, CXCL2, CXCL4, CXCL9, and CXCL10	CD86, CD16, CD32, CD68, iNOS, IL-1R, and MHC-II	Neurotoxicity, oxidative stress, neuronal, and synaptic damage	[21,23]
M2	M2a	IL-4, IL-10, IL-13, and IL-1Ra	CCL24 and CCL22	CD163, CD206, Arg1, Ym-1, FIZZ-1, and MHC-II	Tissue repair, phagocytosis, and encapsulation of parasites	[19,21,24,25]
	M2b	IL-10, IL-1, and IL-6	CCL1	CD86 and MHC-II	Phagocytosis and regulation of inflammatory responses	[21,26]
	M2c	IL-10 and TGF-β	CCL16, CXCL13, and CCR5	CD163, TLR1, and TLR8	Immunoregulation and tissue healing	[21,27]
	M2d	IL-10, IL-12, TNF-α, and TGF-β	CXCL13, CCL16, and CCL18	VEGF	Angiogenesis in tumor	[21]

IL, interleukin; TNF-α, tumor necrosis factor-α; CCL, C-C motif ligand; CXCL, C-X-C motif ligand; CD, cluster of differentiation; iNOS, inducible nitric oxide synthase; MHC-II, major histocompatibility complex-II; Arg1, arginase-1; Ym-1, chitinase-like protein; FIZZ-1, found in inflammatory zone; TGF-β, transforming growth factor-β; CCR, C-C chemokine receptor; TLR, Toll-like receptor; VEGF, vascular endothelial growth factor.

## Data Availability

Not applicable.
